# Supplementary material for: The transcription factor Zeb1 controls homeostasis and function of type 1 conventional dendritic cells
Source: Nat Commun. 2023 Oct 20;14:6639. doi: 10.1038/s41467-023-42428-7 (PMC10589231; doi:10.1038/s41467-023-42428-7)
Supplement: Supplementary file 1 — Supplementary Information [file 41467_2023_42428_MOESM1_ESM.pdf]

Supplementary information for

The transcription factor Zeb1 controls homeostasis and function of type 1 conventional dendritic cells

Yan Wang[1,#], Quan Zhang[2,3,#], Tingting He[1], Yechen Wang[1], Tianqi Lu[1], Zengge Wang[1], Yiyi Wang[1], Shen Lin[3], Kang Yang[1], Xinming Wang[1], Jun Xie[1], Ying Zhou[2,3], Yazhen Hong[1], Wen-Hsien Liu[1], Kairui Mao[1], Shih-Chin Cheng[1], Xin Chen[1], Qiyuan Li[2,3,4,*], Nengming Xiao[1,*]

[1] State Key Laboratory of Cellular Stress Biology, Innovation Center for Cell Signaling Network, School of Life Sciences, Faculty of Medicine and Life Sciences, Xiamen University, Xiamen, Fujian 361102, China

[2] National Institute for Data Science in Health and Medicine, Xiamen University, Fujian 361102, China

[3] School of Medicine, Xiamen University, Xiamen, Fujian 361102, China

[4] Department of Hematology, The First Affiliated Hospital of Xiamen University, Xiamen, 361003, China

# These authors contributed equally to this study: Yan Wang, Quan Zhang.

***Correspondence should be addressed to Q.L. (qiyuan.li@xmu.edu.cn) or N.X. (nengming@xmu.edu.cn).**

Supplementary information includes:

Supplementary Figures 1-9

Legends for Supplementary Data 1-5

Supplementary Figure 1

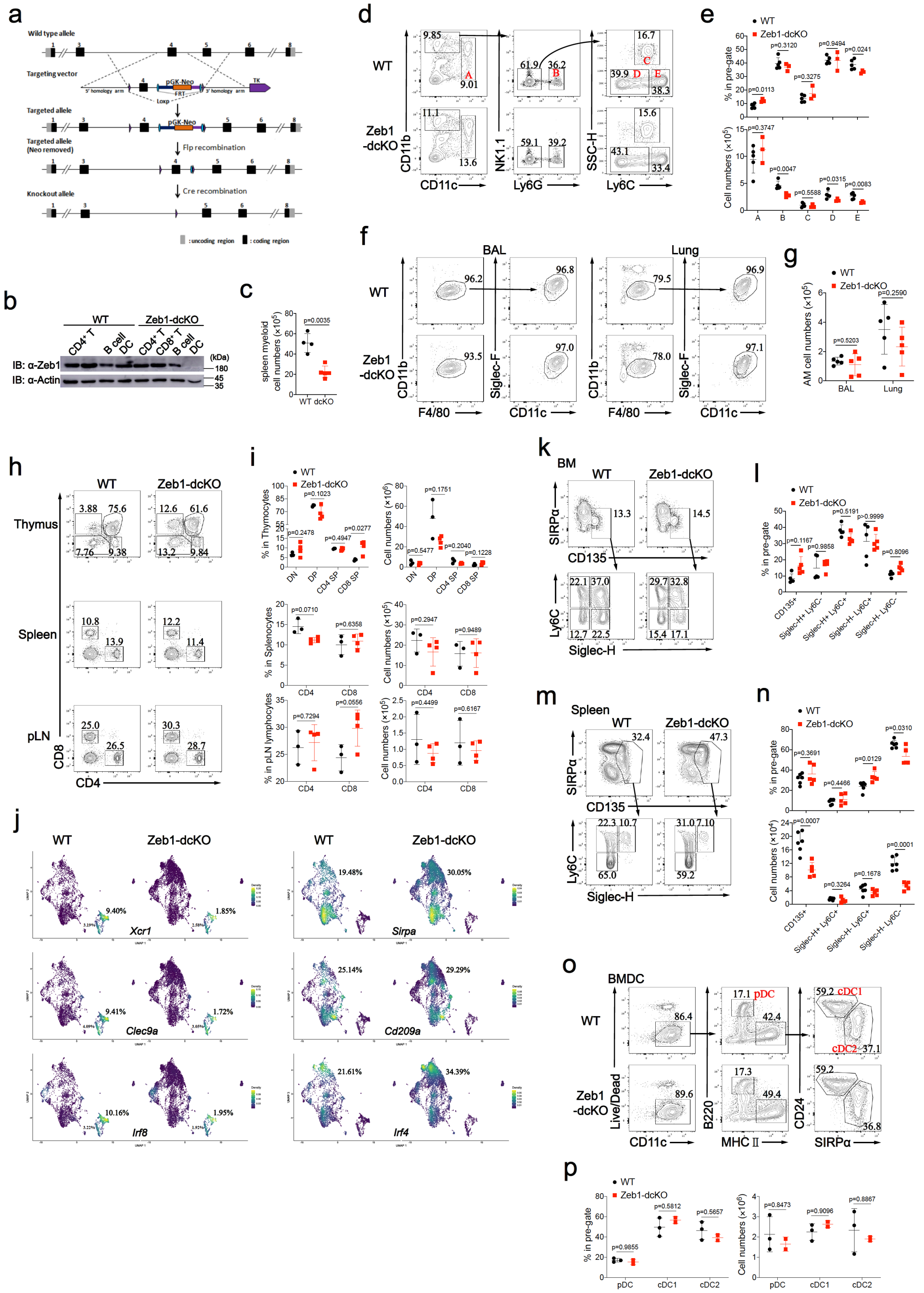

Supplementary Fig. 1. Zeb1 expression in DC is dispensable for the development of immune cells except splenic cDC1.

(**a**) Strategy for Zeb1 gene targeting. (**b**) Immunoblot analysis of Zeb1 protein in immune cells of spleens from WT and Zeb1-dcKO mice. (**c**) Numbers of total myeloid cells in the spleen from WT and Zeb1-dcKO mice (WT, n=4; Zeb1-dcKO, n=5) (**d**) Flow cytometry of Lin^- splenocytes from WT and Zeb1-dcKO mice was used to identify myeloid leukocytes including $CD11c^+$ dendritic cells (A), $CD11b^+Ly6G^+$ neutrophils (B), $CD11b^+Ly6G^-SSC^{hi}$ eosinophils (C), $Ly6C^-$ resident (D) and $Ly6C^+$ inflammatory (E) $CD11b^+Ly6G^-SSC^{lo}$ monocytes/macrophages. (**e**) Frequencies (among their pre-gated cells) and numbers of myeloid leukocytes in the spleen from mice as in **d** (WT, n=5; Zeb1-dcKO, n=3). (**f**) Flow cytometry of $CD45^+$ cells was used to identify alveolar macrophage (AM) in BAL fluid and lung from WT and Zeb1-dcKO mice. (**g**) Numbers of AM ($CD11c^+$Siglec-F^+) in BAL fluid and lung from mice as in **e** (n=5). (**h**) Flow cytometry of thymocytes, splenocytes, and pLN lymphocytes from WT and Zeb1-dcKO mice. (**i**) Frequencies and numbers of thymocyte subsets and peripheral T cells from mice as in **h** (WT, n=3; Zeb1-dcKO, n=4). (**j**) Feature plots demonstrating the density distribution of signature genes expression at single-cell level in WT and Zeb1-deficient splenic cDC populations using sc-RNA-seq data. (**k**, **m**) Flow cytometry of live Lin^- $CD11c^+CD45R^-MHC\ II^{-/int}$ CDP in BM (**k**) or spleen (**m**) from WT and Zeb1-dcKO mice, was used to identify $CD135^+$ pre-DC and their subsets. (**l**, **n**) Frequencies and numbers of $CD135^+$ pre-DC and their subsets among their pre-gated cells in BM (**l:** n=5) or spleen (**n:** WT, n=6; Zeb1-dcKO, n=5) from mice as in **k**. (**o**) Flow cytometry of live $CD11c^+$ Flt3L-cultured BM cells of WT and Zeb1-dcKO mice was used to identify pDC, cDC1 and cDC2. (**p**) Frequencies and numbers of pDC, cDC1 and cDC2 among their pre-gated cells in Flt3L-cultured BM cells as in **k** (WT, n=3; Zeb1-dcKO, n=2). Each symbol represents an individual mouse, small horizontal lines indicate the mean (± s.d.). Data are representative of three independent experiments. Data are presented as mean ± s.d. Statistical analysis was performed using Two-tailed unpaired Student's test. Source data are provided as a Source Data file.

Supplementary Figure 2

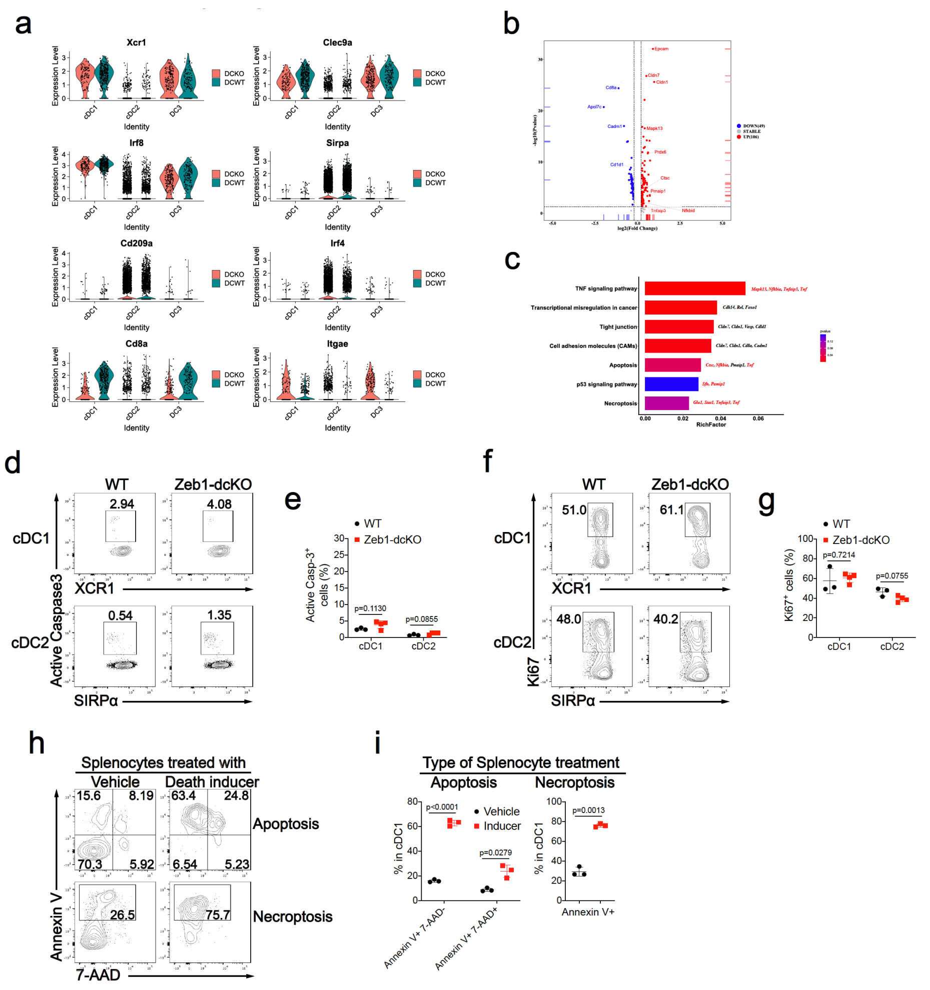

Supplementary Fig. 2. Zeb1 deficiency does not affect identity, proliferation and apoptosis of splenic cDC1.

(**a**) Violin plots showing expression of signature genes in three cDC subsets from spleen of WT and Zeb1-dcKO mice. (**b**) Volcano plot illustrating differentially expressed genes (DEGs) in WT and Zeb1-deficient splenic cDC1 by scRNA-seq analysis. (**c**) KEGG pathway analysis of DEGs in WT and Zeb1-deficient splenic cDC1 by scRNA-seq analysis. (**d**, **f**) Flow cytometry of splenic cDC1 and cDC2 from WT and Zeb1-dcKO mice. (**e**, **g**) Frequencies of active Caspase-3^{+} cells (**e**) or Ki67^{+} cells (**g**) among splenic cDC1 or cDC2 from mice as in **d** (WT, n=3; Zeb1-dcKO, n=4). (**h**) Flow cytometry of splenic cDC1 after coculturing with live, apoptotic, or necroptotic splenocytes (Death inducer: TNF-α+Smac to induce apoptosis, TNF-α+Smac+Zvad to induce necroptosis). (**i**) Frequencies of dying cells among splenic cDC1 as in **h** (n=3). Each symbol represents an individual mouse, small horizontal lines indicate the mean (± s.d.). Data are representative of two (**d** to **i**) independent experiments. Data are presented as mean ± s.d. Statistical analysis was performed using Two-tailed unpaired Student's test. Source data are provided as a Source Data file.

Supplementary Figure 3

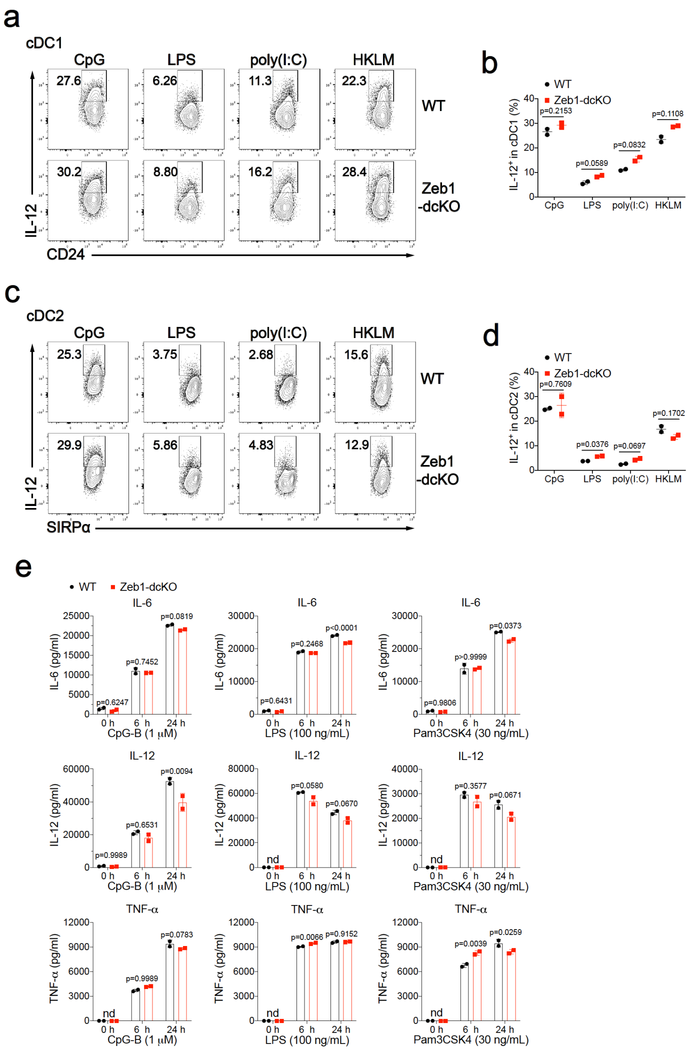

Supplementary Fig. 3. Zeb1-deficient cDC have intact innate immune responses.

(**a**, **c**) Flow cytometry of WT and Zeb1-deficient Flt3L-cDC1 or -cDC2 challenged with different agonists including the TLR4 ligand LPS, TLR9 ligand CpG-B DNA, TLR3 ligand Poly(I:C) or HKLM for 4 h. Numbers adjacent to outlined areas indicate percentage of IL-12-producing cDC1 or cDC2. (**b**, **d**) Frequencies of IL-12-producing cDC1 or cDC2 among Flt3L-cDC1 or -cDC2 as in **a** or **c** (n=2). (**e**) ELISA of cytokines including IL-6, IL-12 and TNF-α in WT and Zeb1-deficient Flt3L-cDC stimulated with CpG-B, LPS or TLR2 ligand Pam3CSK4 for indicated times (n=2). Each symbol represents an individual sample, small horizontal lines indicate the mean (± s.d.). Data are representative of three independent experiments. Data are presented as mean ± s.d. nd, not detected. Statistical analysis was performed using Two-tailed unpaired Student's test. Source data are provided as a Source Data file.

Supplementary Figure 4

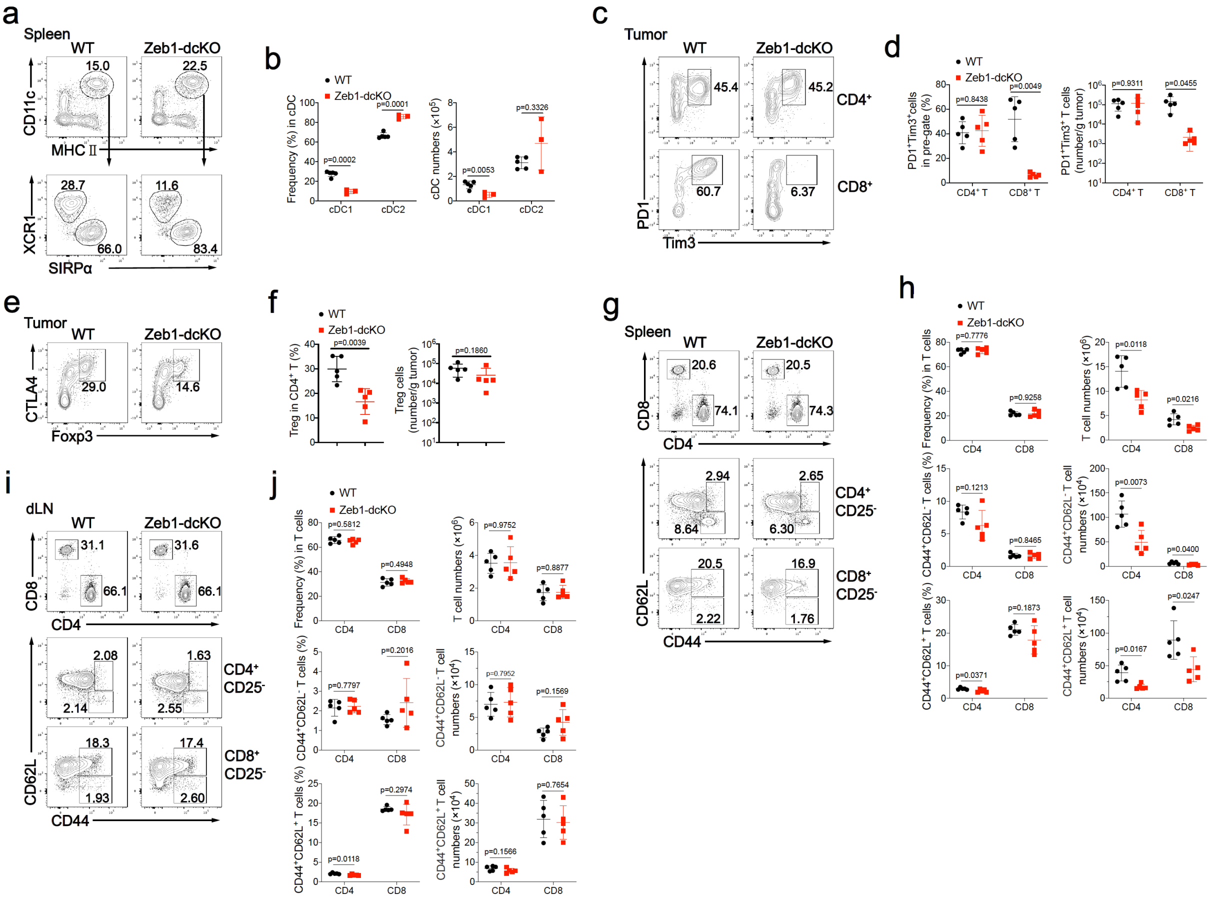

Supplementary Fig. 4. Zeb1 deletion in DC does not affect T cell activation in secondary lymphoid organs of tumor-bearing mice.

(**a**) Flow cytometry of live Lin^-Ly6C^- myeloid cells was used to identify cDC1 ($CD11c^+MHC\ II^+XCR1^+SIRP\alpha^-$) and cDC2 ($CD11c^+MHC\ II^+XCR1^-SIRP\alpha^+$) subsets in spleen from WT and Zeb1-dcKO mice at day 12 after s.c. injection with 2×10^5 B16F10 melanoma cells. (**b**) Frequencies (among total splenic cDC) and numbers of cDC1 and cDC2 in spleen of mice as in **a** (WT, n=5; Zeb1-dcKO, n=3). (**c**) Flow cytometry of activated ($CD45^+TCR\beta^+CD44^+CD62L^-$) $CD4^+$ T cells (top row) and activated $CD8^+$ T cells (bottom row) in tumor from WT and Zeb1-dcKO mice at day 12 after s.c. injection with 2×10^5 B16F10 melanoma cells. (**d**) Frequencies (among activated T cells) and numbers of exhausted ($PD\text{-}1^+Tim3^+$) $CD4^+$ T cells or $CD8^+$ T cells in tumor from mice as in **c** (n=5). (**e**) Flow cytometry of ($CD45^+TCR\beta^+$) $CD4^+$ T cells in tumors from mice as in c. (**f**) Frequencies (among $CD4^+$ T cells) and numbers of ($Foxp3^+CTLA4^+$) Treg cells in tumor from mice as in **e** (n=5). (**g**, **i**) Flow cytometry of total T cells (top row), $CD25^-CD4^+$ (middle row) and $CD25^-CD8^+$ (bottom row) T cells in spleen (**g**) or tumor dLN (**i**) from WT and Zeb1-dcKO mice at day 12 after s.c. injection with 2×10^5 B16F10 melanoma cells. **(h**, **j)** Frequencies and numbers of $CD4^+$ or $CD8^+$ T cells (among total T cells) (top row), $CD44^+CD62L^-$ effector memory $CD4^+$ or $CD8^+$ T cells (middle row) and $CD44^+CD62L^+$ central memory $CD4^+$ or $CD8^+$ T cells (bottom row) (among $CD25^-CD4^+$ or $CD25^-CD8^+$ T cells) in spleen (**h**) or tumor dLN (**j**) from mice as in **c** (n = 5). Each symbol represents an individual mouse, small horizontal lines indicate the mean (± s.d.). Data are representative of three independent experiments. Data are presented as mean ± s.d. Statistical analysis was performed using Two-tailed unpaired Student's test. Source data are provided as a Source Data file.

Supplementary Figure 5

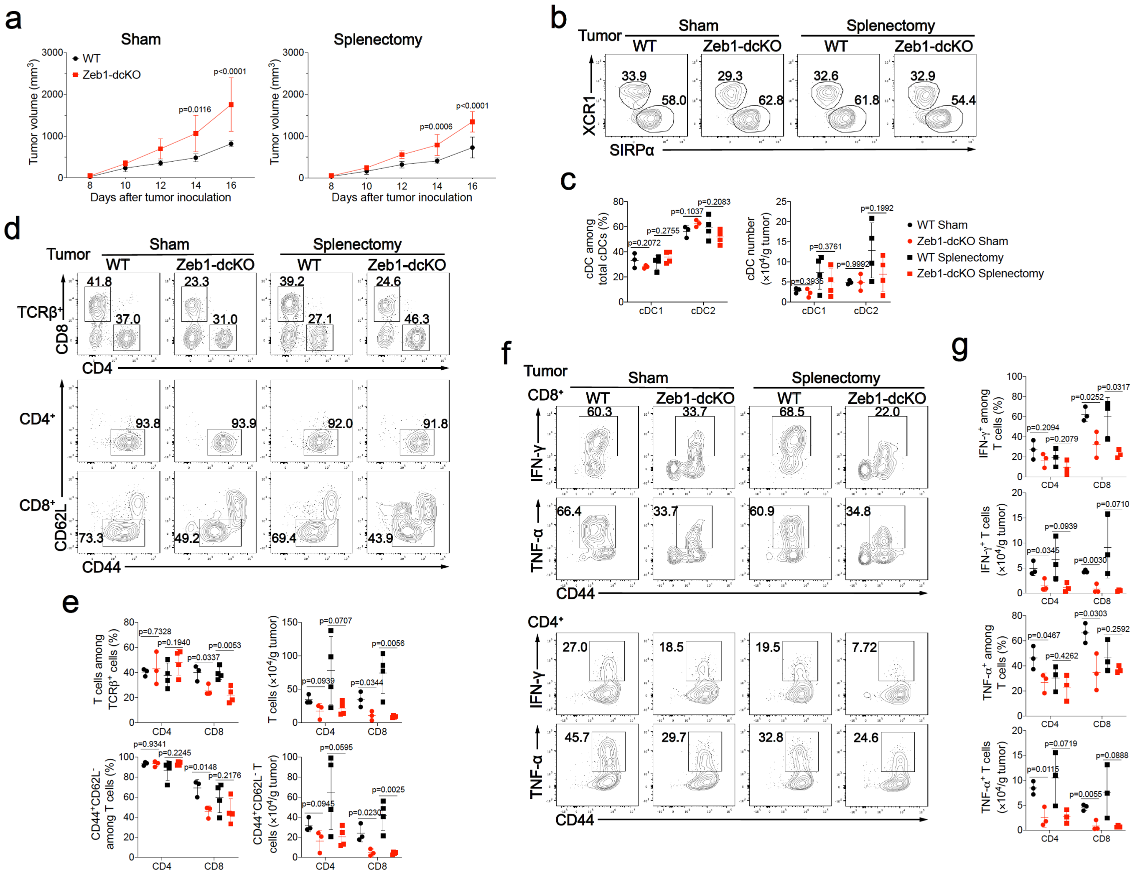

Supplementary Fig. 5. Splenectomy did not ameliorate the defective antitumor T cell response in DC-specific Zeb1-deficient mice.

(a) Tumor growth of WT and Zeb1-dcKO mice injected subcutaneously (s.c.) with 2×10^5 B16F10 melanoma cells 6 days after splenectomy or sham-operation (n=5). (**b**) Flow cytometry of Lin^- $CD45^+CD64^-F4/80^-MHC\ II^+CD11c^+CD26^+$ myeloid cells was used to identify cDC1 ($XCR1^+SIRP\alpha^-$) and cDC2 ($XCR1^-SIRP\alpha^+$) subsets in tumors from mice as in **a** at day 16 after tumor inoculation. (**c**) Frequencies and numbers of cDC1 and cDC2 among $CD11c^+CD26^+$ cDCs in tumors of mice as in **b** (sham, n=3; splenectomy, n=4). (**d**) Flow cytometry of tumor infiltrating $TCR\beta^+$ T cells (TITs) (top row), TIL $CD4^+$ T cells (middle row), TIL $CD8^+$ T cells (bottom row) in tumor from mice as in **b.** (**e**) Frequencies and numbers of TIL $CD4^+$ or $CD8^+$ T cells (among TITs) (top row) and $CD44^+CD62L^-$ $CD4^+$ (among TIL $CD4^+$ T cells) or $CD44^+CD62L^-$ $CD8^+$ (among TIL $CD8^+$ T cells) activated T cells (bottom row) in tumor from mice as in **b** (sham, n=3; splenectomy, n=4). (**f**) Flow cytometry of TIL $CD8^+$ T cells (Top row), TIL $CD4^+$ T cells (bottom rows) in tumor from mice as in **b**. (**g**) Frequencies and numbers of IFN-γ- and TNF-α-producing $CD8^+$ T cells (among TIL $CD8^+$ T cells) and of IFN-γ- and TNF-α-producing $CD4^+$ T cells (among TIL $CD4^+$ T cells) in tumor from mice as in **b** (n=3). Each symbol represents an individual mouse, small horizontal lines indicate the mean (± s.d.). Data are presented as mean ±s.d. Statistical analysis was performed using two-tailed unpaired Student's t-test (**c**, **e**, **g**), or two-way ANOVA with Sidak's multiple comparisons test (**a**). Source data are provided as a Source Data file.

Supplementary Figure 6

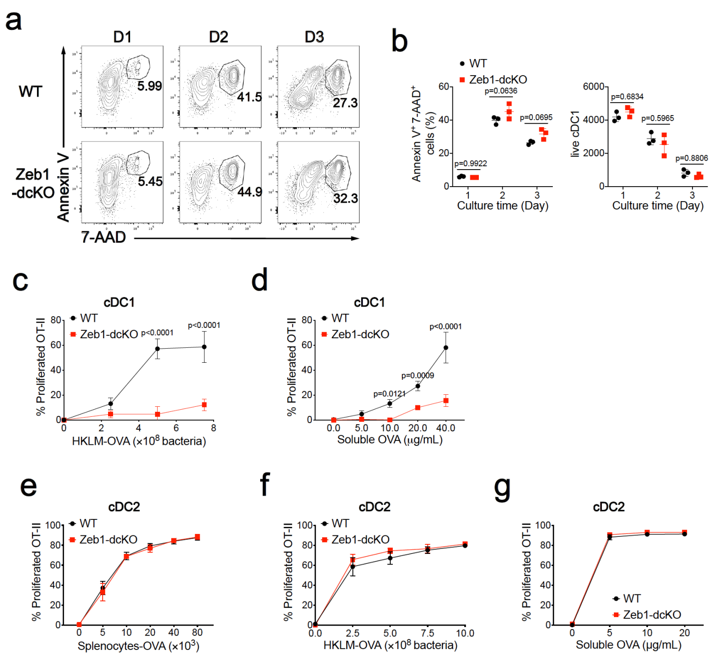

Supplementary Fig. 6. Zeb1 is required for presentation of cell-associated and soluble antigens through MHC-Ⅱ by cDC1 but not by cDC2.

(**a**) Flow cytometry of the indicated Flt3L-cDC1 after being cultured for indicated days with CFSE-labeled OT-Ⅰ T cells and HKLM-OVA. Numbers adjacent to outlined areas indicate percent dying (Annexin V^{+} 7-AAD^{+}) Flt3L-cDC1. (**b**) Frequencies of dying Flt3L-cDC1 among total cDC1 at different days from **a** (n=3). Each symbol represents an individual sample, small horizontal lines indicate the mean (± s.d.). (**c,d**) WT and Zeb1-deficient Flt3L-cDC1 were cultured for 3 days with CFSE-labeled OT-Ⅱ T cells and different dose of HKLM-OVA (**c**) or soluble OVA (**d**) respectively, and assayed for OT-Ⅱ proliferation and activation (CFSE^{-}CD44^{+}). (**e** to **g**) Flt3L-cDC2 of both genotypes were cultured for 3 days with CFSE-labeled OT-Ⅱ T cells and various dose of irradiated OVA-loaded β2m$^{-/-}$ splenocytes (**e**), or HKLM-OVA (**f**), or soluble OVA (**g**) respectively, and assayed for OT-Ⅱ proliferation and activation (CFSE^{-}CD44^{+}). Data are representative of three independent experiments. Data are presented as mean ± s.d. Statistical analysis was performed using two-way ANOVA with Sidak's multiple comparisons test. Source data are provided as a Source Data file.

Supplementary Figure 7

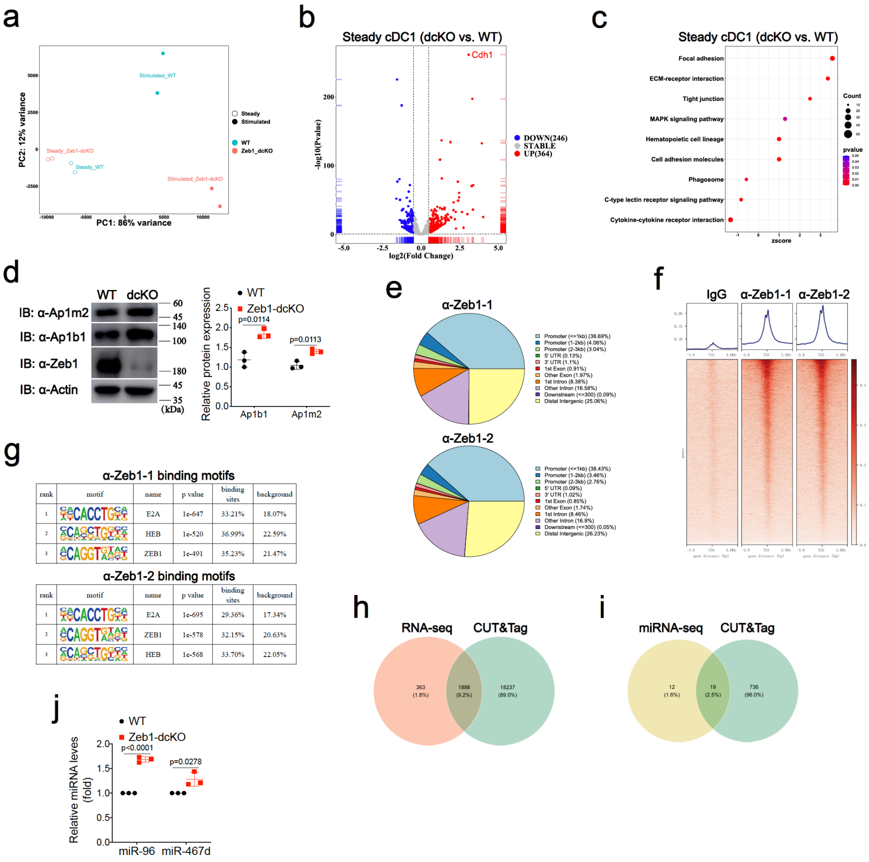

Supplementary Fig. 7. Integrated analysis of transcriptomes and Zeb1 occupancy identifies Zeb1 target genes that support cross-presentation.

(**a**) Principal Component Analysis (PCA) of the transcriptomes of WT and Zeb1-deficient Flt3L-cDC1 at steady state and after stimulation with HKLM-OVA for 4 h. (**b**) Volcano plots illustrating DEGs in WT and Zeb1-deficient Flt3L-cDC1 at steady state (n=2). (**c**) Bubble plot depicting KEGG pathway analysis of DEGs in WT and Zeb1-deficient Flt3L-cDC1 at steady state (n=2). (**d**) Immunoblot analysis of Ap1b1 and Ap1m2 in WT and Zeb1-deficient Flt3L-cDC1 after stimulation with HKLM-OVA for 4 h. Relative protein level is quantified by normalization to actin protein (n=3). (**e**) Pie charts demonstrating the distribution of Zeb1 binding peaks (CUT&Tag) across the genome annotated by ChIPseeker from independent biological replicates. (**f**) Deposition of Zeb1 binding peaks (CUT&Tag) centered on the promoter regions (TSS ± 3 kb) from independent biological replicates. (**g**) Known motif analysis identifying three top enriched DNA motifs from Zeb1 binding peaks (CUT&Tag) of independent biological replicates using HOMER database. (**h**) Venn diagram illustrating intersection of DEGs by comparing RNA-seq with CUT&Tag of Flt3L-cDC1 after stimulation. (**i**) Venn diagram illustrating intersection of DEGs by comparing miRNA-seq with CUT&Tag of Flt3L-cDC1 after stimulation. (**j**) Quantitative realtime PCR demonstrating relative expression of miR-96 and miR-467d in WT and Zeb1-deficient Flt3L-cDC1 after stimulation with HKLM-OVA for 4 h. Each symbol represents an individual sample, small horizontal lines indicate the mean (± s.d.). Data are representative of three independent experiments. Data are presented as mean ± s.d. Statistical analysis was performed using Two-tailed unpaired Student’s test. Source data are provided as a Source Data file.

Supplementary Figure 8

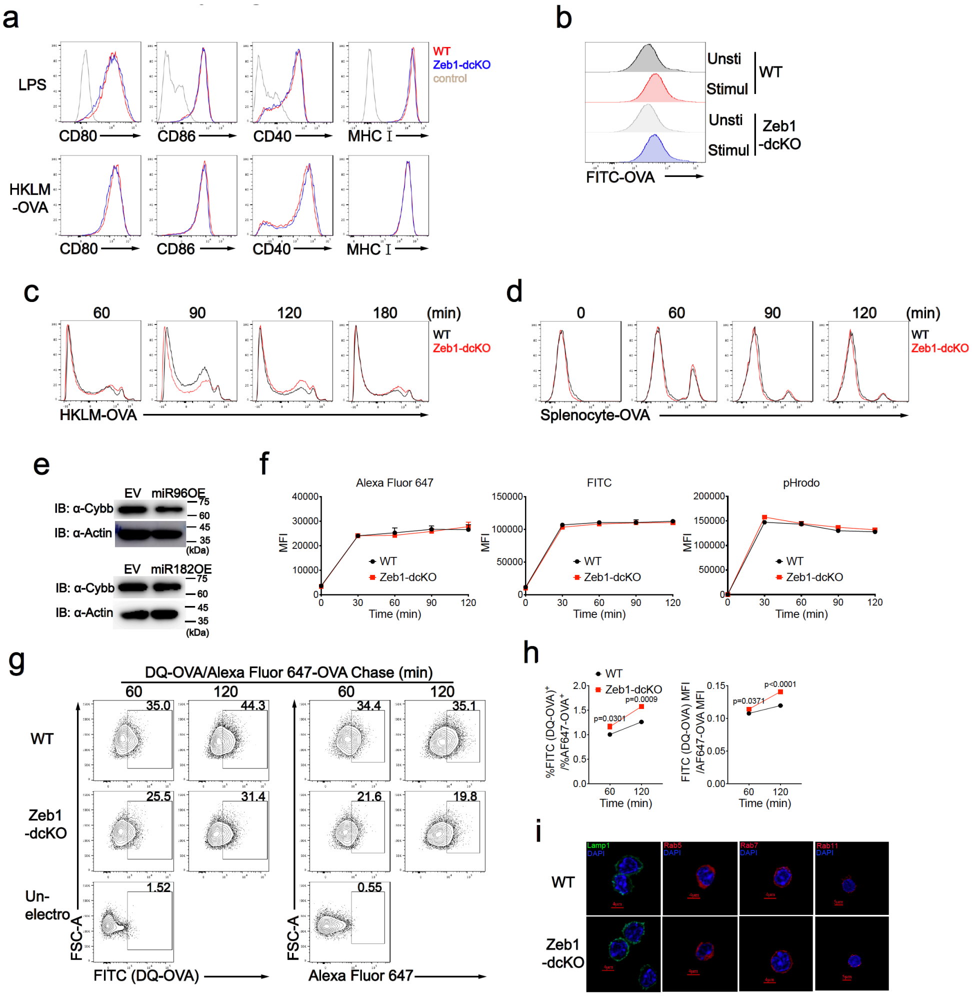

Supplementary Fig. 8. Zeb1 ablation in DC does not affect cDC1 maturation and phagocytosis.

(**a**) Flow cytometry of WT and Zeb1-deficient Flt3L-cDC1 challenged with 1 μg/ml LPS or 1×10^8 CFU HKLM-OVA for 24 h to measure the expression of CD80, CD86, CD40 and MHC-Ⅰ. (**b**) Flow cytometry of WT and Zeb1-deficient Flt3L-cDC1 incubated with or without Alexa Fluor 488-labelled OVA for 4 h. (**c**, **d**) Flow cytometry of WT and Zeb1-deficient Flt3L-cDC1 incubated with Alexa Fluor 647-labelled HKLM-OVA (**c**) or Alexa Fluor 488-labelled OVA-loaded $\beta 2m^{-/-}$ splenocyte (Splenocyte-OVA) (**d**) for indicted times.(**e**) Immunoblot analysis of Cybb in purified Flt3L-cDC1 transduced with control retrovirus (EV) or retrovirus encoding miR-96 (miR96OE) (top row) or miR-182 (miR182OE) (bottom row) during Flt3L-induced in-vitro DC development. (**f**) WT and Zeb1-deficient Flt3L-cDC1 were cultured with 1×10^8 CFU Alexa Fluor 647-labelled, or FITC-labelled, or pHrodo-labelled HKLM-OVA for the indicated times. The mean intensity of indicated fluorescence was analyzed by flow cytometry to reflect the change of phagosomal pH. (**g** and **h**) DQ-OVA/Alexa Fluor 647-OVA were introduced into the cytosols of WT and Zeb1-deficient Flt3L-cDC1 by electroporation, and cytosolic antigen degradation were measured by flow cytometric analysis after chasing for indicated time (**g**). The cytosolic antigens degradation was quantified as the ratio of FITC $(DQ\text{-}OVA)^+$ cells to Alexa Fluor 647^+ cells (**h**, left) or the ratio of MFI of FITC (DQ-OVA) fluorescence to MFI of Alexa Fluor 647 fluorescence (**h**, right). (**i**) Confocal microscope images of steady WT and Zeb1-deficient Flt3L-cDC1 stained with anti-Lamp1, anti-Rab5, anti-Rab7 or anti-Rab11 antibodies. Data are representative of three independent experiments. Data are presented as mean ± s.d. Statistical analysis was performed using two-tailed unpaired Student's t-test (**h**), or two-way ANOVA with Sidak's multiple comparisons test (**f**). Source data are provided as a Source Data file.

Supplemental Figure 9 (to be continued)

a

cDC and pDC in lymphoid tissues

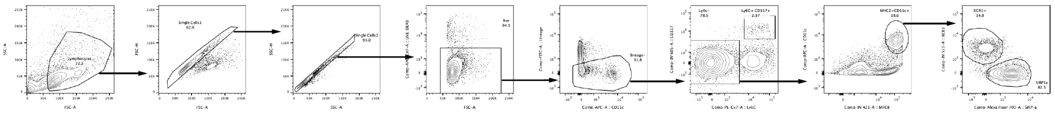

cDC in non-lymphoid tissues

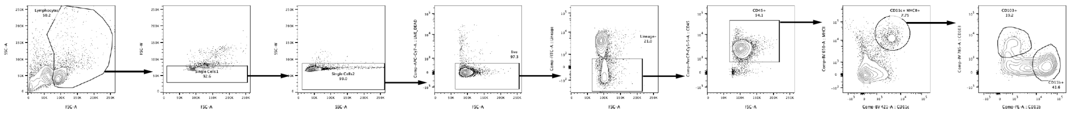

b

Cell death of cDC in lymphoid tissues

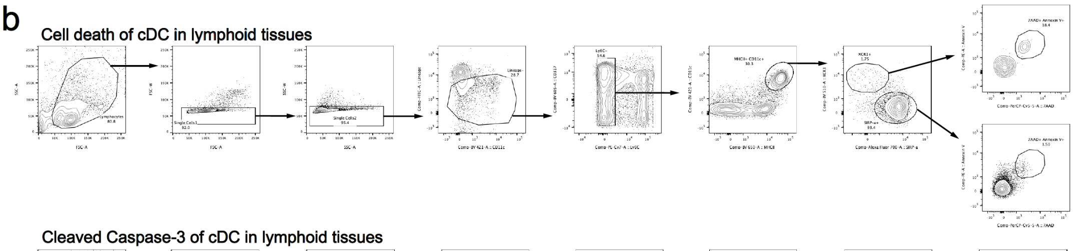

Cleaved Caspase-3 of cDC in lymphoid tissues

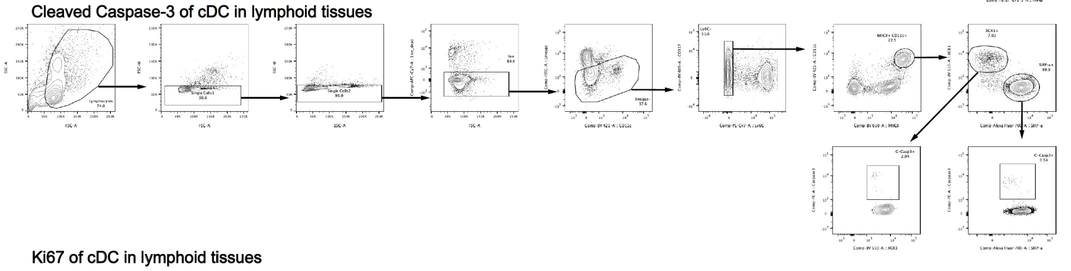

Ki67 of cDC in lymphoid tissues

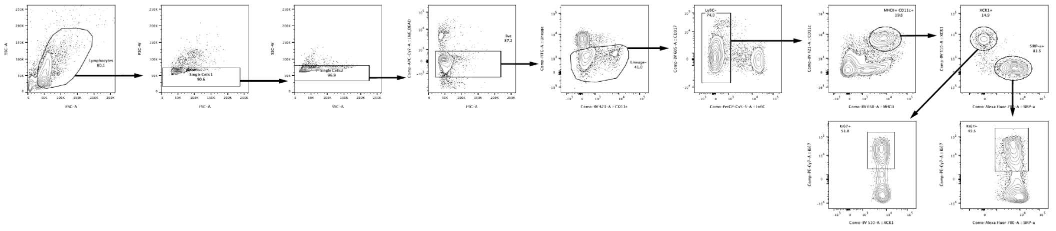

Supplemental Figure 9 (continued)

c

Myeloid cells in spleen

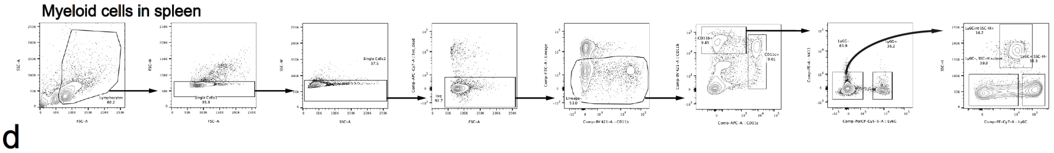

d

Alveolar macrophages in BAL fluid and lung

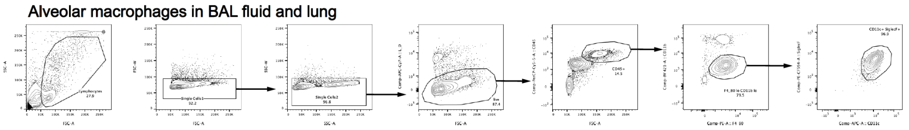

e

Pre-DC in bone marrow

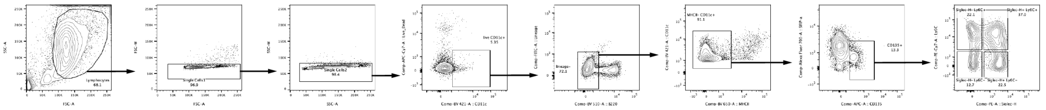

Pre-DC in spleen

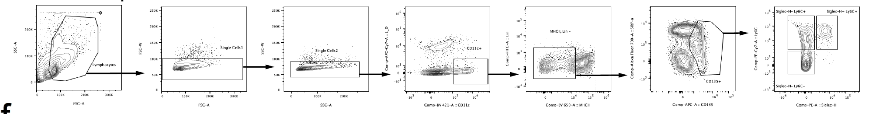

f

in vitro DC development

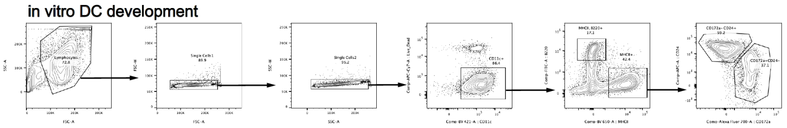

g

T cells in spleen and LNs

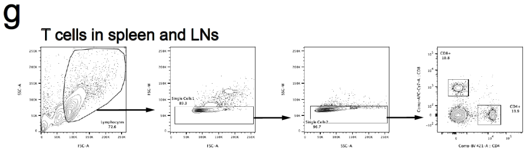

T cells in Thymus

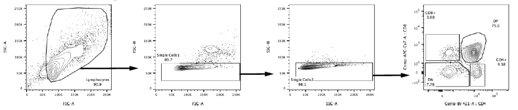

Supplemental Figure 9 (continued)

h

cDC in spleen of mixed bone marrow chimeric mice

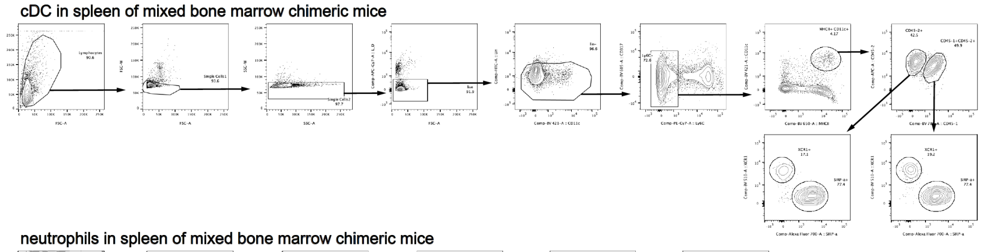

neutrophils in spleen of mixed bone marrow chimeric mice

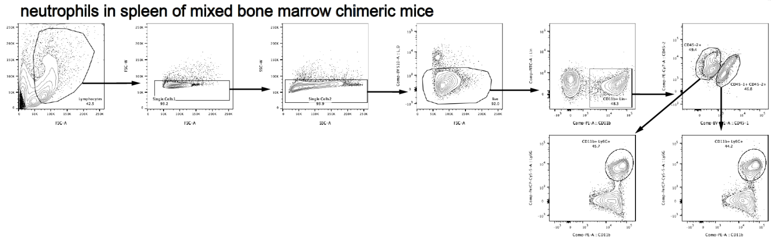

i

cDC in B16F10 tumors from tumor-bearing mice

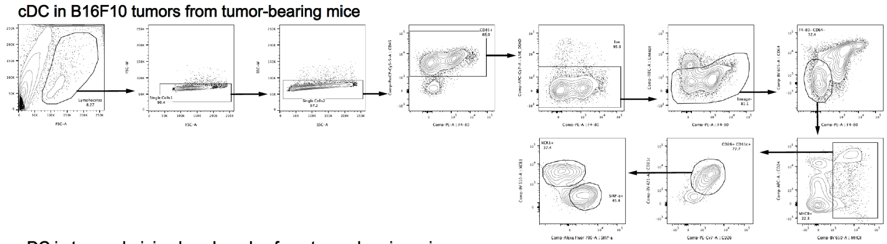

cDC in tumor draining lymph nodes from tumor-bearing mice

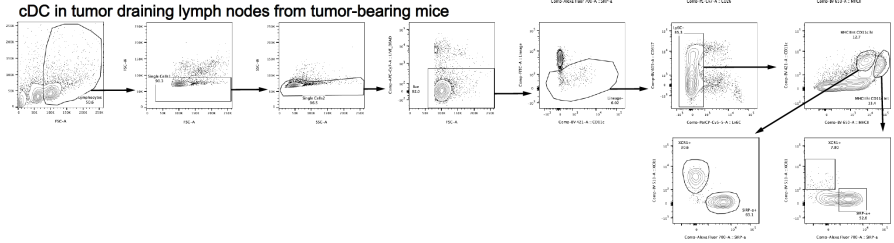

Supplemental Figure 9 (continued)

j

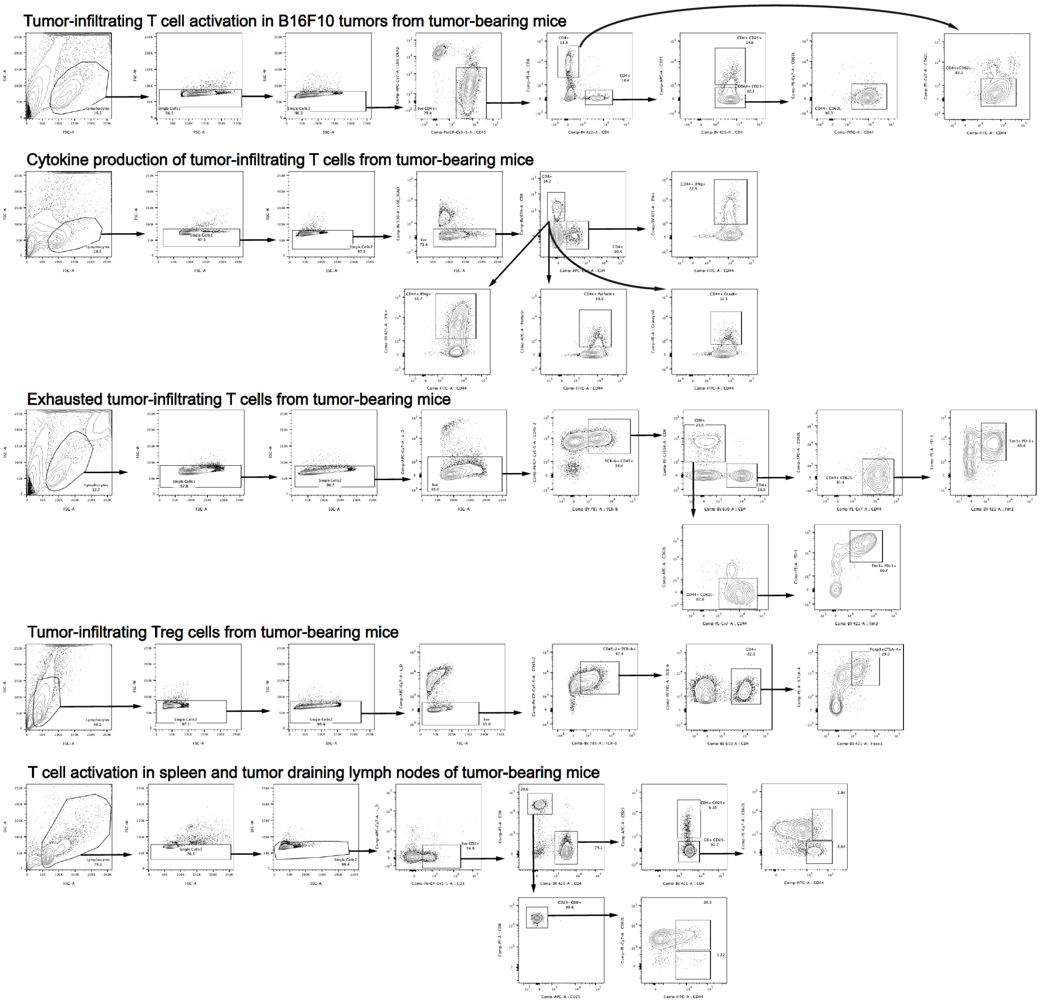

Supplemental Figure 9 (continued)

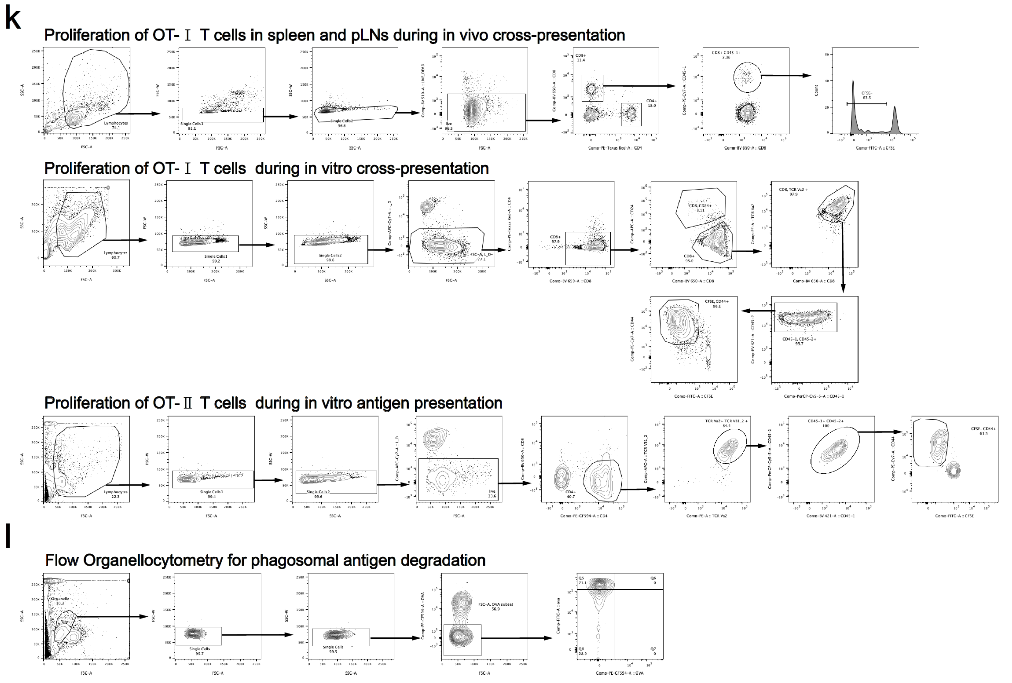

Supplementary Fig. 9. Gating strategies of flow cytometric analysis.

(a) Representative flow cytometric plots showing gating strategy for cDC and pDC subsets in lymphoid tissues and non-lymphoid tissues. (**b**) Representative flow cytometric plots showing gating strategy for cell death, cleaved caspase-3 or Ki67 of cDC in lymphoid tissues. (**c**) Representative flow cytometric plots showing gating strategy for myeloid cells in spleen. (**d**) Representative flow cytometric plots showing gating strategy for alveolar macrophages in BAL fluid and lung. (**e**) Representative flow cytometric plots showing gating strategy used to identify pre-DC subsets in bone marrow and spleen. (**f**) Representative flow cytometric showing gating strategy for DC subsets in Flt3L-cultured BM cells. (**g**) Representative flow cytometric plots showing gating strategy for T cells in lymphoid tissues. (**h**) Representative flow cytometric plots showing gating strategy for cDC or neutrophils in spleen of mixed bone marrow chimeric mice. (**i**) Representative flow cytometric S plots showing gating strategy for cDC subsets in B16F10 tumors and tumor dLNs from tumor-bearing mice. (**j**) Representative flow cytometric plots showing the gating strategy for T cell activation, cytokine production, exhaustion and Treg population of tumor-infiltrating T cells, and for T cell activation in spleen and tumor dLNs, of tumor-bearing mice. (**k**) Representative flow cytometric plots showing gating strategy for proliferation of OT-I or OT-II T cells during in vivo cross-presentation or in vitro cross-presentation. (**l**) Representative flow cytometric plots showing gating strategy for phagosomal antigen degradation.

Supplementary Data

Supplementary Data 1. Single-Cell Resolution Reveals Disruptions in Cell Population Composition and Differential Positive Gene Expression Ratios in splenic cDC Samples (WT vs. Zeb1-dcKO).

Supplementary Data 2. Insights into RNA-Seq data from steady Flt3L-cDC1 (WT vs. Zeb1-dcKO): Summary and Interpretation of Analysis Findings.

Supplementary Data 3. Insights into RNA-Seq Data from Flt3L-cDC1 (WT vs. Zeb1-dcKO) upon stimulation with HKLM-OVA for 4 hours: Summary and Interpretation of Analysis Findings.

Supplementary Data 4. Unveiling Insights from Comprehensive Analysis of Small RNA-Seq Data from Flt3L-cDC1 (WT vs. Zeb1-dcKO) upon stimulation with HKLM-OVA for 4 hours.

Supplementary Data 5. The information of all antibodies used in this study for cell purification, for flow cytometry, for immunofluorescence, for western blot and for ELISA.
