# Supplementary material for: The transcription factor Zeb1 controls homeostasis and function of type 1 conventional dendritic cells
Source: Nat Commun. 2023 Oct 20;14:6639. doi: 10.1038/s41467-023-42428-7 (PMC10589231; doi:10.1038/s41467-023-42428-7)
Supplement: Supplementary file 3 — Description of Additional Supplementary Files [file 41467_2023_42428_MOESM3_ESM.pdf]

Description of Additional Supplementary Files

File Name: Supplementary Data 1
Description: Single-Cell Resolution Reveals Disruptions in Cell Population Composition and Differential Positive Gene Expression Ratios in splenic cDC Samples (WT vs. Zeb1-dcKO).

File Name: Supplementary Data 2
Description: Insights into RNA-Seq data from steady Flt3L-cDC1 (WT vs. Zeb1-dcKO): Summary and Interpretation of Analysis Findings.

File Name: Supplementary Data 3
Description: Insights into RNA-Seq Data from Flt3L-cDC1 (WT vs. Zeb1-dcKO) stimulated with HKLM-OVA for 4 hours: Summary and Interpretation of Analysis Findings.

File Name: Supplementary Data 4
Description: Unveiling Insights from Comprehensive Analysis of Small RNA-Seq Data from Flt3LcDC1 (WT vs. Zeb1-dcKO) stimulated with HKLM-OVA for 4 hours.

File Name: Supplementary Data 5
Description: The information of all antibodies used in this study for cell purification, for flow cytometry, for immunofluorescence, for western blot and for ELISA.
