# Supplementary material for: The transcription factor Zeb1 controls homeostasis and function of type 1 conventional dendritic cells
Source: Nat Commun. 2023 Oct 20;14:6639. doi: 10.1038/s41467-023-42428-7 (PMC10589231; doi:10.1038/s41467-023-42428-7)
Supplement: Supplementary file 5 — Reporting Summary [file 41467_2023_42428_MOESM5_ESM.pdf]

nature portfolio

Corresponding author(s): Qiyuan Li; Nengming Xiao

Last updated by author(s): Sep 19, 2023

Reporting Summary

Nature Portfolio wishes to improve the reproducibility of the work that we publish. This form provides structure for consistency and transparency in reporting. For further information on Nature Portfolio policies, see our Editorial Policies and the Editorial Policy Checklist.

Statistics

For all statistical analyses, confirm that the following items are present in the figure legend, table legend, main text, or Methods section.

n/a	Confirmed	
☐	☒	The exact sample size (*n*) for each experimental group/condition, given as a discrete number and unit of measurement
☐	☒	A statement on whether measurements were taken from distinct samples or whether the same sample was measured repeatedly
☐	☒	The statistical test(s) used AND whether they are one- or two-sided<br>*Only common tests should be described solely by name; describe more complex techniques in the Methods section.*
☒	☐	A description of all covariates tested
☐	☒	A description of any assumptions or corrections, such as tests of normality and adjustment for multiple comparisons
☐	☒	A full description of the statistical parameters including central tendency (e.g. means) or other basic estimates (e.g. regression coefficient) AND variation (e.g. standard deviation) or associated estimates of uncertainty (e.g. confidence intervals)
☐	☒	For null hypothesis testing, the test statistic (e.g. *F*, *t*, *r*) with confidence intervals, effect sizes, degrees of freedom and *P* value noted<br>*Give P values as exact values whenever suitable.*
☒	☐	For Bayesian analysis, information on the choice of priors and Markov chain Monte Carlo settings
☒	☐	For hierarchical and complex designs, identification of the appropriate level for tests and full reporting of outcomes
☒	☐	Estimates of effect sizes (e.g. Cohen's *d*, Pearson's *r*), indicating how they were calculated

Our web collection on statistics for biologists contains articles on many of the points above.

Software and code

Policy information about availability of computer code

Data collection

Flow cytometry : BD fortessa X-20; Real-Time PCR:Bio-RAD CFX96; scRNA-seq:the NextSeq illumina Platform ;
Bulk RNA-seq: Illumina NovaSeq platform; miRNA-seq: Illumina NovaSeq 6000 platform; Cut&Tag: Illumina Nova-PE150;
Confocal images: Leica TCS SP8 DLS and Zeiss LSM 900+Airyscan2 confocal microscopes.

Data analysis

Statistics and Data plotting: GraphPad Prism 8.0; Flow cytometry: Flowjo version 10.8.1; R softvare(http://www.project.org): R version 4.1.3
scRNA-seq: Raw data were filtered using fastp. The clean data based on UMI was mapped to Mus musculus genome (mm10) utilizing STAR. Seurat package (version 3.0) in R was used to perform normalization. Cell type clusters were further identified in PCA by FindClusters.
Bulk RNA-seq :Raw data (raw reads) of Fastq format were removed adapter to yield clean data. The clean data (clean reads) were mapped to Mus musculus reference genome (mm10) using Hisat2 (version 2.0.5) for each sample. Uniquely mapped reads were counted by FeatureCounts (version 1.5.0-p3). The counts matrix was normalized with size factors using DESeq2. Differential expression analysis of two conditions/groups (two biological replicates per condition) was performed using the DESeq2 R package. Differential expressed genes were found using DESeq2 with cutoffs abs (log2FC > 0.5). Gene set enrichment analysis were performed using clusterProfiler R package;
miRNA-seq: Raw data (raw reads) of Fastq format were firstly processed through SHRiMP2, a short-read mapping program. The micro RNA reads were processed by Bowtie2 version 2.3.4 for read mapping to Mus musculus reference sequence (mm10). To identify conserved miRNAs, the predicted miRNA hairpins were compared against miRNA precursor sequences from miRBase22.0 using mirDeep2 (version 2.0.0.5) and srna-tools-cli (http://srna-tools.cm.uea.ac.uk/) were used to obtain the potential miRNA and draw the secondary structures. MirDeep2 quantifier.pl was used to obtain the miRNA counts, and custom scripts were used to obtain base bias on the first position of identified miRNA with certain length and on each position of all identified miRNA respectively. Target gene prediction of miRNA was performed by the TargetScan (version 8.0) database;
CUT&Tag: Paired-end reads were aligned to Mus musculus reference genome (mm10) using Bowtie2 version 2.3.4 with options: -end-to-end -

very-sensitive -no-mixed --no-discordant --phred33 -I 10 -X 700 -p 2. For maximum economy, up to 96 barcoded samples per 2-lane flow cell could be pooled for 25×25 bp sequencing. For peak calling, parameters which been used were macs2 callpeak -t input -g mm -f BAMPE -n –outdir output --broad --broad-cutoff 0.1 -B --SPMR --keep-dup all -n out_name. DESeq2 was applied to differential peak analysis.
Real-time PCR data : Bio-Rad CFX Manager software (version: 1.0.1035.131).

For manuscripts utilizing custom algorithms or software that are central to the research but not yet described in published literature, software must be made available to editors and reviewers. We strongly encourage code deposition in a community repository (e.g. GitHub). See the Nature Portfolio guidelines for submitting code & software for further information.

Data

Policy information about availability of data

All manuscripts must include a data availability statement. This statement should provide the following information, where applicable:

- Accession codes, unique identifiers, or web links for publicly available datasets
- A description of any restrictions on data availability
- For clinical datasets or third party data, please ensure that the statement adheres to our policy

Data availability
scRNA-seq, RNA-seq, CUT&tag and miRNA-seq datasets that support the findings of this study have been deposited in the Gene Expression Omnibus (GEO) (https://www.ncbi.nlm.nih.gov/geo/query/acc.cgi) with accession numbers GSE208311, GSE216059, GSE206963, GSE206964, GSE207161, respectively. All data needed to evaluate the conclusions in the paper are present in the paper and/or the Supplementary Materials. Source data are provided with the paper.

Human research participants

Policy information about studies involving human research participants and Sex and Gender in Research.

Reporting on sex and gender	N/A.
Population characteristics	N/A.
Recruitment	N/A.
Ethics oversight	N/A.

Note that full information on the approval of the study protocol must also be provided in the manuscript.

Field-specific reporting

Please select the one below that is the best fit for your research. If you are not sure, read the appropriate sections before making your selection.

☒ Life sciences ☐ Behavioural & social sciences ☐ Ecological, evolutionary & environmental sciences

For a reference copy of the document with all sections, see nature.com/documents/nr-reporting-summary-flat.pdf

Life sciences study design

All studies must disclose on these points even when the disclosure is negative.

Sample size	The sample size for each experiment is indicated in the figure legends. For in vivo studies, at least 5 mice per group are sufficient to detect meaningful biological differences. For in vitro studies, all the experiments were replicated at least for 3 biological replicated or at least two or three independent repeats.
Data exclusions	No data was excluded from this study.
Replication	Both in vitro and in vivo experiments were performed with at least 3 biological replicated or at least two or three independent repeats.
Randomization	Littermate mice were randomly grouped into control and treatment groups for all experiments in this study. Age and gender-matched mice between 8-10 weeks were used across groups.
Blinding	No blinding was involved in experiments in this paper. Researchers were aware of genotyping results prior to experiments. Genotyping of mice was conducted by an independent person.<br>Investigators were not blinded during data acquisition and data analysis, because the same data acquisition settings and data analysis settings (compensation and gating) were applied to all samples of an experiment in flow cytometric data analysis precluding a subjective influence of knowledge of the sample name and belonging to sample groups on the data.

Reporting for specific materials, systems and methods

We require information from authors about some types of materials, experimental systems and methods used in many studies. Here, indicate whether each material, system or method listed is relevant to your study. If you are not sure if a list item applies to your research, read the appropriate section before selecting a response.

Materials & experimental systems

n/a	Involved in the study
☐	☒ Antibodies
☐	☒ Eukaryotic cell lines
☒	☐ Palaeontology and archaeology
☐	☒ Animals and other organisms
☒	☐ Clinical data
☒	☐ Dual use research of concern

Methods

n/a	Involved in the study
☐	☒ ChIP-seq
☐	☒ Flow cytometry
☒	☐ MRI-based neuroimaging

Antibodies

Antibodies used

Biotin-conjugated antibodies for cell purification: anti-mouse F4/80 (1:200 dilution; Clone#BM8; Cat#123106), anti-mouse NK1.1 (1:200 dilution; Clone#PK136; Cat#108704), anti-mouse TER-119 (1:200 dilution; Clone#TER-119; Cat#116204), anti-mouse CD3ε (1:100 dilution; Clone#145-2C11; Cat#100304), anti-mouse CD19 (1:100 dilution; Clone#6D5; Cat#115504), anti-mouse CD4 (1:100 dilution; Clone#GK1.5; Cat#100404), anti-mouse CD8α (1:100 dilution; Clone#53-6.7; Cat#100704), anti-mouse B220 (1:100 dilution; Clone#RA3-6B2; Cat#103204), anti-mouse CD25 (1:200 dilution; Clone#PC61; Cat#102004), anti-mouse CD44 (1:200 dilution; Clone#IM7; Cat#103004), anti-mouse CD11b (1:200 dilution; Clone#M1/70; Cat#101204), anti-mouse CD11c (1:200 dilution; Clone#N418; Cat#117304), anti-mouse Gr-1 (1:200 dilution; Clone#RB6-8C5; Cat#108404), anti-mouse TCRγ/δ (1:200 dilution; Clone#GL3; Cat#118103) and purified anti-mouse CD16/32 (1:100 dilution; Clone#93; Cat#101302) were purchased from BioLegend. For flow cytometry: anti-mouse CD317 (1:200 dilution; Clone#927; Cat#127025), anti-mouse Ly-6C (1:200 dilution; Clone#HK1.4; Cat#128018), anti-mouse XCR1 (1:200 dilution; Clone#ZET; Cat#148218), anti-mouse F4/80 (1:200 dilution; Clone#BM8; Cat#123110), anti-mouse Siglec-H (1:200 dilution; Clone#551; Cat#129606), anti-mouse CD172α (SIRP-α) (1:200 dilution; Clone#P84; Cat#144022), anti-mouse CD26 (DPP-4) (1:200 dilution; Clone#H194-112; Cat#137810), anti-mouse CD135 (1:200 dilution; Clone#A2F10; Cat#135310), anti-mouse CD115 (1:200 dilution; Clone#AFS98; Cat#135517), anti-mouse CD62L (1:200 dilution; Clone#MEL-14; Cat#104441), anti-mouse CD64 (1:200 dilution; Clone# X54-5/7.1; Cat#139323), anti-mouse TCR Vα2 (1:200 dilution; Clone#B20.1; Cat#127807), anti-mouse Granzyme B (1:200 dilution; Clone#GB11; Cat#515403), anti-mouse CD45R/B220 (1:400 dilution; Clone#RA3-6B2; Cat#103211 and 103205), anti-mouse IFN-γ (1:200 dilution; Clone#XMG1.2; Cat#505810), anti-mouse TCR Vβ5.1/.2 (1:200 dilution; Clone#MR9-4; Cat#139506), anti-mouse Perforin (1:200 dilution; Clone#S16009A; Cat#154303), anti-mouse H2Kb (1:200 dilution; Clone#AF6-88.5; Cat#116517), anti-mouse CD366 (Tim-3) (1:200 dilution; Clone#RMT3-23; Cat#119723), anti-mouse CD279 (PD-1) (1:200 dilution; Clone#29F.1A12; Cat#135205), anti-mouse CD152 (CTLA-4) (1:200 dilution; Clone#UC10-4B9; Cat#106305), and anti-mouse Ki67 (1:200 dilution; Clone#Ki-67; Cat#350503) were purchased from BioLegend. Anti-mouse Ly-6G (1:200 dilution; Clone#1A8; Cat#551460), anti-mouse CD24 (1:200 dilution; Clone#M1/69; Cat#562349), anti-mouse CD45R/B220 (1:400 dilution; Clone#RA3-6B2; Cat#563103, 562290, 563894, 563893 and 563708), anti-mouse CD44 (1:200 dilution; Clone#IM7; Cat#560569), anti-mouse Foxp3 (1:200 dilution; Clone#FJK-16s; Cat#77-5775-40), anti-mouse MHC II (I-A/I-E) (1:400 dilution; Clone#M5/114.15.2; Cat#563415) and anti-mouse CD8α (1:200 dilution; Clone#53-6.7; Cat#563234, 562283 and 553033) were purchased from BD Biosciences. Anti-mouse CD8α (1:200 dilution; Clone#53-6.7; Cat#48-0081-82), anti-mouse CD45.1 (1:200 dilution; Clone#A20; Cat#48-0453-82), anti-mouse CD4 (1:200 dilution; Clone# GK1.5 or RM4-5; Cat#17-0041-83, 47-0042-82 and 48-0042-82), anti-mouse CD11c (1:200 dilution; Clone#N418; Cat#48-0114-82), anti-mouse CD117 (c-Kit) (1:200 dilution; Clone#2B8; Cat#12-1171-81), anti-mouse CD19 (1:200 dilution; Clone#1D3; Cat#11-0193-85), anti-mouse CD25 (1:200 dilution; Clone#PC61.5; Cat#17-0251-82), anti-mouse CD3ε (1:200 dilution; Clone#145-2C11; Cat#11-0031-85), anti-mouse CD45.2 (1:200 dilution; Clone#104; Cat#48-0454-82) and anti-mouse TNF-α (1:200 dilution; Clone#TN3-19.12; Cat#12-7423-41) were purchased from eBioscience. Annexin V Apoptosis detection Kit (Cat#559763) and Active Caspase-3 Apoptosis Kit (Cat#550914) were from BD Biosciences. Fixable Viability Dye eFluorTM 780 (1:400 dilution; Cat#65-0865-18) was purchased from eBioscience. For Western blots: anti-β-actin (1:1000 dilution; Clone#8H10D10; Cat#3700), Rabbit IgG (1:5000 dilution; Clone#DA1E; Cat#3900) Isotype Control were purchased from Cell Signaling Technology; anti-Zeb1 (1:1000 dilution; Cat#21544-1-AP) was from Proteintech; anti-Cybb (1:1000 dilution; Cat#A19701) and anti-Ncf2 (1:1000 dilution; Cat#A3703) were purchased from ABclonal. For immunofluorescence: anti-Rab5 Rabbit mAb (1:200 dilution; Clone#C8B1; Cat#3547T), anti-Rab7 Rabbit mAb (1:200 dilution; Clone#D95F2; Cat#9367T) and anti-Lamp1 Mouse mAb (1:200 dilution; Clone#D4O1S; Cat#15665T) were from Cell Signaling Technology; Anti-Galectin-3 Alexa Fluor 488 (1:200 dilution; Clone#A3A12; Cat#sc-53127 AF488) was from Santa Cruz Biotechnology.

Validation

All antibodies were well-recognized clones in the field, commercially available and validated by the manufacturers.

Eukaryotic cell lines

Policy information about cell lines and Sex and Gender in Research

Cell line source(s)

HEK293T were from American Type Culture Collection center (ATCC).

Authentication

HEK293T cells were pre-authenticated by ATCC by STR sequencing.

Mycoplasma contamination	No cell lines were positive for Mycoplasma contamination in a PCR test method.
Commonly misidentified lines (See ICLAC register)	No commonly misidentified lines were used.

Animals and other research organisms

Policy information about studies involving animals; ARRIVE guidelines recommended for reporting animal research, and Sex and Gender in Research

Laboratory animals	All mice were maintained in specific pathogen-free facilities of Xiamen University Laboratory Animal Center. All animal protocols were approved by members of the Institutional Animal Care and Use Committee of Xiamen University. C57BL/6J (B6) (#000664), B6.SJL (#002014), OT- Ⅰ (#003831), OT- Ⅱ (#004194), β2m-/- (#002087), CD11c-Cre (#008068) mice were originally from the Jackson Laboratory. Zeb1fl/fl (C57BL/6J-Zeb1tm1(flox-Neo)Smoc) mice was generated by Shanghai Model Organisms Center, Inc.
Wild animals	No wild animals were used in the study.
Reporting on sex	No reporting on sex.
Field-collected samples	No field-collected samples were employed in this study.
Ethics oversight	All the mice were housed in a specific pathogen-free facility under a 12h light-dark cycle at the Xiamen University Laboratory Animal Center. All animal protocols were approved by members of the Institutional Animal Care and Use Committee of Xiamen University.

Note that full information on the approval of the study protocol must also be provided in the manuscript.

ChIP-seq

Data deposition

☒ Confirm that both raw and final processed data have been deposited in a public database such as GEO.

☐ Confirm that you have deposited or provided access to graph files (e.g. BED files) for the called peaks.

Data access links *May remain private before publication.*	CUT&tag datasets have been deposited in the Gene Expression Omnibus (GEO) (https://www.ncbi.nlm.nih.gov/geo/query/acc.cgi) with accession numbers GSE206964.
Files in database submission	GSE208311, GSE216059, GSE206963, GSE206964, GSE207161
Genome browser session (e.g. UCSC)	https://www.ncbi.nlm.nih.gov/geo/query/acc.cgi?acc=GSE206963<br>https://www.ncbi.nlm.nih.gov/geo/query/acc.cgi?acc=GSE206964<br>https://www.ncbi.nlm.nih.gov/geo/query/acc.cgi?acc=GSE207161<br>https://www.ncbi.nlm.nih.gov/geo/query/acc.cgi?acc=GSE208311<br>https://www.ncbi.nlm.nih.gov/geo/query/acc.cgi?acc=GSE216059

Methodology

Replicates	duplicate.
Sequencing depth	Libraries were sequenced on an Illumina Nova-PE150 and 150-bp paired-end reads were generated.
Antibodies	anti-Zeb1 antibody (Cat#21544-1-AP; Proteintech) or Rabbit IgG Isotype Control (Cat#3900; Cell Signaling Technology).
Peak calling parameters	For peak calling, parameters which been used were macs2 callpeak -t input -g mm -f BAMPE -n –outdir output --broad --broad-cutoff 0.1 -B --SPMR --keep-dup all -n out_name.
Data quality	Paired-end Illumina sequencing was analyzed based on the barcoded libraries following the manufacturer's description. Paired-end reads were aligned to Mus musculus reference genome (mm10) using Bowtie2 version 2.3.4 with options: -end-to-end -very-sensitive -no-mixed --no-discordant --phred33 -I 10 -X 700 -p 2. For maximum economy, up to 96 barcoded samples per 2-lane flow cell could be pooled for 25×25 bp sequencing.
Software	DESeq2 was applied to differential peak analysis.

Flow Cytometry

Plots

Confirm that:

☒ The axis labels state the marker and fluorochrome used (e.g. CD4-FITC).

☒ The axis scales are clearly visible. Include numbers along axes only for bottom left plot of group (a 'group' is an analysis of identical markers).

☒ All plots are contour plots with outliers or pseudocolor plots.

☒ A numerical value for number of cells or percentage (with statistics) is provided.

Methodology

Sample preparation	For blocking of Fc receptors, single cell suspensions were incubated with purified anti-CD16/32 for 15 min on ice prior to immunostaining. Surfaces of cells in suspensions were stained with fluorochrome-conjugated antibodies in flow cytometry buffer (0.5% BSA and 0.05% NaN3 in PBS) for 30 min at 4°C. To monitor apoptosis, cells were fixed and permeabilized with Cytofix/Cytoperm buffer after cell surface staining. Cells were then stained with anti-active Caspase-3 antibody (Cat#550914, BD Biosciences) in Perm/Wash buffer (1×). Intracellular cytokines were stained after stimulation of cells for 4 h with HKLM-OVA or phorbol 12-Myristate 13-Acetate (PMA, 50 ng/ml; Sigma-Aldrich) plus ionomycin (1 µg/ml; Sigma-Aldrich) in the presence of GolgiStop (Cat#554724, BD Biosciences). Cells were incubated with antibodies against cell surface markers, and then fixed and permeabilized with Cytofix/Cytoperm buffer (Cat#51-2090KZ, BD Biosciences). Cells were then stained with antibodies against indicated cytokines. All flow cytometry data were acquired on BD LSRFortessa and were analyzed using FlowJo software (BD Biosciences).<br>For cell sorting, cell suspensions were stained with fluorochrome-conjugated antibodies in sorting buffer (1 mM EDTA, 25 mM HEPES, 1% FBS in PBS) and then sorted with BD Aria sorter. Splenic or LN DCs were sorted as L/D-Lin-MHC Ⅱ +CD11c +CD317-Ly6C- (cDCs), L/D-Lin-MHC Ⅱ +CD11c+CD317-Ly6C-XCR1+SIRP-α-(cDC1s). Flt3L-cDC1s were sorted as L/D-B220-MHC Ⅱ +CD11c+CD24+SIRP-α- and Flt3L-cDC2s were sorted as L/D-B220-MHC Ⅱ +CD11c+CD24-SIRP-α+.
Instrument	BD fortessa X-20, BD FACSAria Fusion
Software	Flowjo v 10.8.1
Cell population abundance	Cells were sorted using BD FACSAria Fusion.
Gating strategy	Gating strategies for all cell populations are described within the manuscript.

☒ Tick this box to confirm that a figure exemplifying the gating strategy is provided in the Supplementary Information.
