# Supplementary figures and images for: The transcription factor Zeb1 controls homeostasis and function of type 1 conventional dendritic cells
Source: Nat Commun. 2023 Oct 20;14:6639. doi: 10.1038/s41467-023-42428-7 (PMC10589231; doi:10.1038/s41467-023-42428-7)

Fig 5g

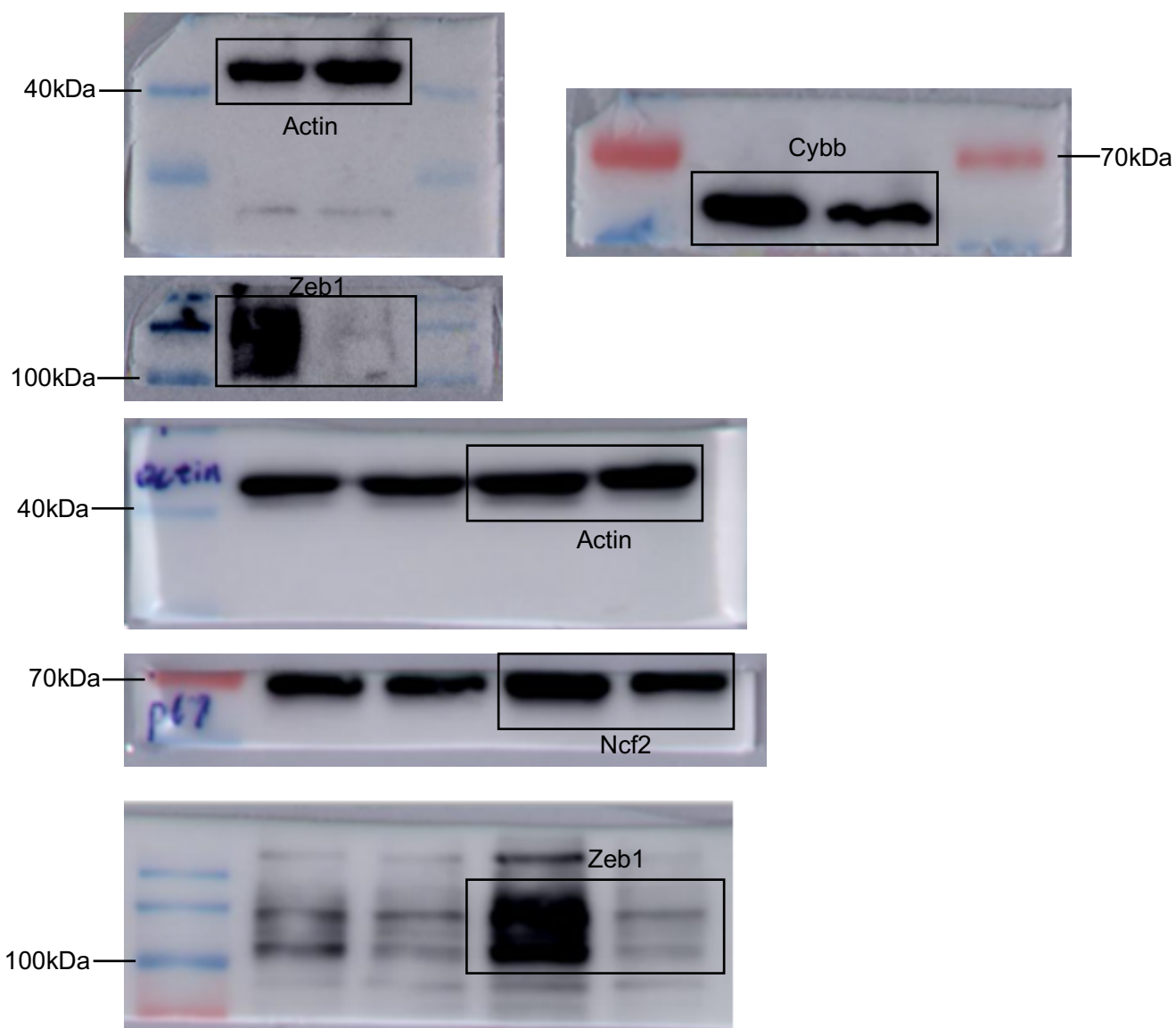

Fig S1b

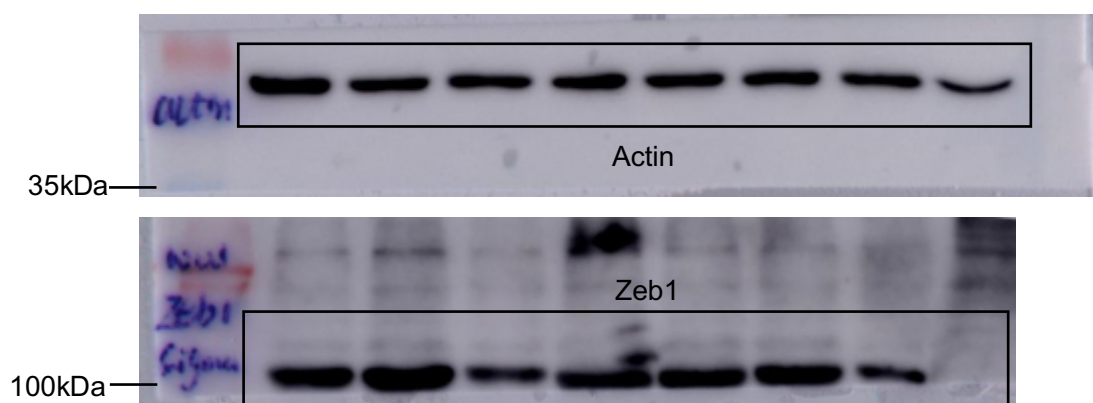

Fig S7d

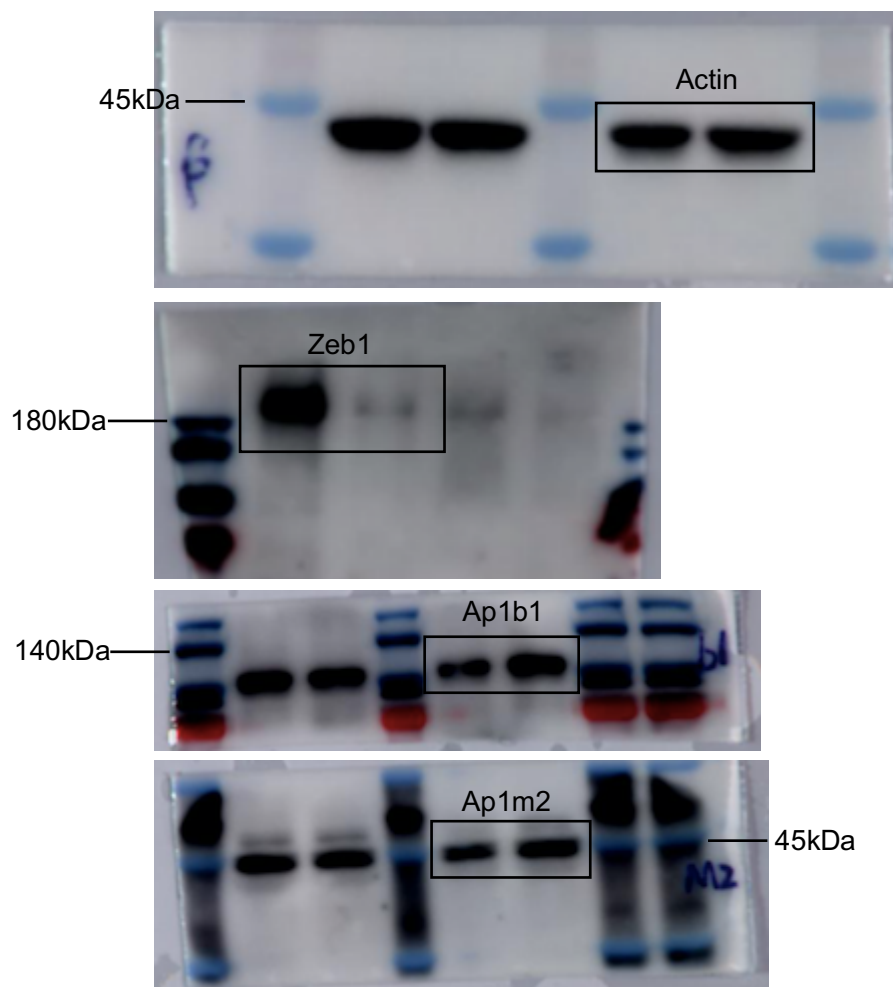

Fig S8e

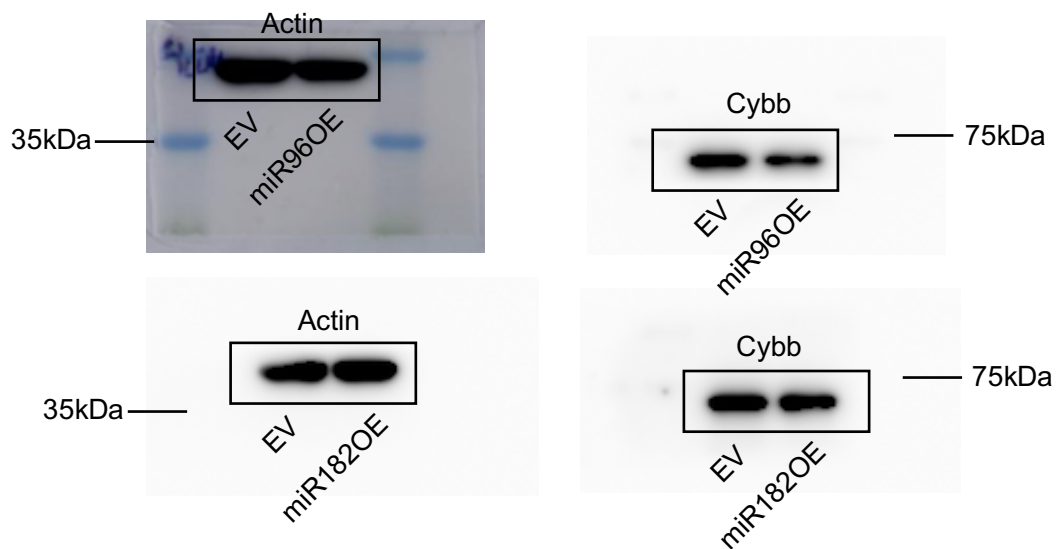

Supplement: Supplementary file 6 — Source Data [file 41467_2023_42428_MOESM6_ESM.zip › Uncropped WB.pdf]
